# Measured Hyperelastic Properties of Cervical Tissue with Shear-Wave Elastography

**DOI:** 10.3390/s22010302

**Published:** 2021-12-31

**Authors:** Weirong Ge, Graham Brooker, Ritu Mogra, Jon Hyett

**Affiliations:** 1 Faculty of Medicine and Health, University of Sydney, Camperdown, NSW 2006, Australia; 2 Australian Centre for Field Robotics, Rose Street Bldg, University of Sydney, Camperdown, NSW 2006, Australia; g.brooker@acfr.edu.au; 3 Royal Prince Alfred Hospital, 50 Missenden Rd., Camperdown, NSW 2050, Australia; ritu.mogra@health.nsw.gov.au (R.M.); jon.hyett@health.nsw.gov.au (J.H.)

**Keywords:** shear-wave elastography, hyperelastic materials, cervical tissue

## Abstract

The nonlinear mechanical behaviour of cervical tissue causes unpredictable changes in measured elastograms when pressure is applied. These uncontrolled variables prevent the reliable measurement of tissue elasticity in a clinical setting. Measuring the nonlinear properties of tissue is difficult due to the need for both shear modulus and strain to be taken simultaneously. A simulation-based method is proposed in this paper to resolve this. This study describes the nonlinear behaviour of cervical tissue using the hyperelastic material models of Demiray–Fung and Veronda–Westmann. Elastograms from 33 low-risk patients between 18 and 22 weeks gestation were obtained. The average measured properties of the hyperelastic material models are: Demiray–Fung—$A_{1}\alpha$ = 2.07 (1.65–2.58) kPa, α = 6.74 (4.07–19.55); Veronda–Westmann—$C_{1}C_{2}$ = 4.12 (3.24–5.04) kPa, $C_{2}$ = 4.86 (2.86–14.28). The Demiray–Fung and Veronda–Westmann models performed similarly in fitting to the elastograms with an average root mean square deviation of 0.41 and 0.47 ms${}^{-1}$, respectively. The use of hyperelastic material models to calibrate shear-wave speed measurements improved the consistency of measurements. This method could be applied in a large-scale clinical setting but requires updated models and higher data resolution.

## 1. Introduction

The use of Shear-wave Elastography (SWE) has garnered significant interest in the medical community. The ability to quantitatively measure tissue elasticity has been used with different levels of success to evaluate pathologies in various fields. Some examples include evaluating liver fibrosis [1,2], differentiating between benign and malignant breast lesions [3,4], and evaluating tendon injury [5,6].

In obstetrics, there is interest in using the elasticity of cervical tissue as a diagnostic tool. The elasticity of the cervical tissue changes throughout the pregnancy to accommodate the changes in its function. The initial role of the cervix is to provide mechanical support to the fetus. At the point of labour, the elasticity of the cervix decreases drastically to allow the passage of the fetus through the birth canal without causing excessive tissue damage. Measurements of cervical elasticity could predict success in the induction of labour [7] and predict preterm birth [8,9].

Most commercial SWE equipment reports the shear-wave speed or elasticity calculated from a linearly elastic model. These measurements assume that the elasticity of the cervix is constant, but this assumption disagrees with the current research that describes soft tissue to exhibit nonlinear stress–strain behaviour. Multiple studies have noted that this is a significant issue in SWE measurements [6,10]. The nonlinear behaviour of tissue causes errors in the measurements made since, in most clinical settings, the pressure from ultrasound probes is unregulated. This phenomenon, known as “tissue stiffening” or “strain stiffening”, is present in most soft tissues, for example, breast tissue [11], heel pad [12,13], calf muscle [14] and thyroid gland [15]. The occurrence of tissue stiffening is especially detrimental for cervical elastography as the probe is in direct contact with the tissue, and the shape of the probe causes non-uniform deformation [16]. In prior studies, researchers have used acoustoelasticity theory and hyperelastic material models to describe the nonlinear behaviour of tissue mathematically. In most of these experiments, researchers use a controlled pressure or indentation to deform the tissue of interest. They then measure the change in shear-wave speed at the controlled deformation to fit the parameters of the material model [17,18,19]. However, measuring the nonlinear properties of cervical tissue using these methods in the current clinical environment is impractical. Cervical tissue is not as exposed as the other tissues, and the probe cannot be modified to put uniaxial pressure on the tissue.

The overall objective of this work is to generate a practical framework to measure the nonlinear properties of cervical tissue using shear-wave elastography in a clinical environment and use it to calibrate the “tissue stiffening” effects. This pilot study accomplishes three aims: Firstly, the implementation of a simulation-based method of determining the material parameters of cervical tissue—secondly, evaluating the fit of two hyperelastic materials in describing cervical tissue behaviour. Finally, determining the necessary changes to the method and equipment for a future large scale study to establish a diagnosis protocol.

## 2. Theory

The theory of large acoustoelastic effect relates shear-wave speed to tissue deformation. The derivation is detailed in a paper by Ogden [20]. This section provides a basic summary and its use in this context. Hyperelastic material models describe the elastic behaviour of the materials with strain energy density functions. These material functions provide a method to model the nonlinear stress–strain behaviour observed in materials such as soft tissue. The strain energy density function relates strain energy density (Ψ) in the material to the deformation gradient (*F*)). The deformation gradient describes the change of the material from an initial reference configuration to the current configuration as shown in Equation (1)—χ maps the reference configuration (*X*) to a new deformed configuration.

(1) $$F=\frac{\partial \chi }{\partial X}$$

The stress and elasticity of hyperelastic materials are described as a function of the right Cauchy–Green tensor (*C*), which relates to the deformation gradient in Equation (2). $I_{1}$, $I_{2}$ and $I_{3}$ are the invariants of the right Cauchy–Green tensor and can be related to the principal stretches of the deformation gradient ($\lambda_{1,2,3}$), as shown in Equations (3)–(5). The principal stretches are the eigenvalues of the deformation gradient.

(2) $$C=F^{T}F$$

(3) $$I_{1}=\lambda_{1}^{2}+\lambda_{2}^{2}+\lambda_{3}^{2}$$

(4) $$I_{2}=\lambda_{1}^{2}\lambda_{2}^{2}+\lambda_{1}^{2}\lambda_{3}^{2}+\lambda_{2}^{2}\lambda_{3}^{2}$$

(5) $$I_{3}=\lambda_{1}^{2}\lambda_{2}^{2}\lambda_{3}^{2}$$

The material elastic tensor (C) for the deformed configuration relates to the shear-wave speed ($c_{s}$) and the density of the material (ρ) in Equation (6). For this paper, ρ was assumed to be approximately 1 g/cm${}^{3}$ for ease of conversion.

(6) $$\rho {c_{s}}^{2}=C=2\lambda_{2}^{2}\left(\frac{\partial \Psi }{\partial I_{1}}+\frac{\partial \Psi }{\partial I_{2}}\lambda_{2}^{-2}\lambda_{1}^{-2}\right)$$

The hyperelastic materials chosen in this study are the Demiray–Fung (DF) and Veronda–Westmann (VW) models. These models are phenomenological models designed to describe the stress–strain behaviour of soft biological tissue and skin, respectively [21,22,23]. The strain energy density functions for DF and VW are summarised in Table 1. This study aims to describe the material properties of the cervical tissue effectively in a clinical environment with minimal resources. Due to time restrictions in the hospital, the sonographer only took two frames in the same plane of view to measure the tissue’s elasticity. These material models DF and VW have the minimum amount of parameters needed to describe the elastogram with the given restrictions.

The two types of parameters of each material will be referred to as the base and stiffening parameters. The base parameters are the products $A_{1}\alpha$ and $C_{1}C_{2}$. They describe the base speed of the material. This paper defines the base speed as the shear-wave speed in the undeformed reference state, while the measured shear-wave is the shear-wave speed in the current configuration. The stiffening parameters α and $C_{2}$ describe the proportional change in the base speed depending on the deformation of the tissue.

## 3. FEBio Simulation

**Figure 1:**
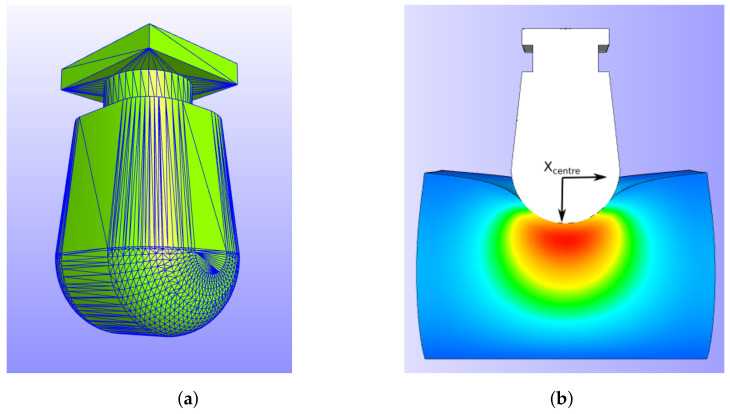
Figures of the simulation in FEBio. (**a**) 3D scan of Toshiba probe used in this study, and (**b**) sample indentation frame with the centre of the probe marked.

Due to the irregular shape of the ultrasound probe, the deformation caused by the probe is uneven across the cervix. The unique changes in the shear-wave speed of the tissue caused by the probe shape and compression magnitude can be simulated and matched to the elastogram to determine the tissue properties. Consequently, in this study, the authors used FEBio [24] to simulate the compression. The 3D scanned CAD model of the ultrasound probe is used to indent a block of nearly incompressible tissue (52 × 40 × 35 mm) as a static structural mechanics problem. Figure 1 shows the simulation, and Table 2 describes the simulation elements. The indentation range applied was 0 to 20 mm.

This study defines shear-wave speed in the tissue with no deformation as the base speed ($c_{s0}$) of the tissue. The principal stretches for each tissue element within the ultrasound frame of view are taken for every indentation step in the simulation. At each indentation, the principal stretches cause a proportional increase in the base speed of the tissue. The proportional-increase (*P*) from the base speed ($c_{s0}$) for stiffening parameters within the range 0.01<α<100 and $0.01<C_{2}<100$ is calculated. This is shown in Equations (7)–(9). The proportional increase field is the increase from the base speed value within the area of interest and is not dependent on the base parameter.

(7) $$P=c_{s}/c_{s0}$$

(8) $$P_{DF}=\lambda_{2}\sqrt{e^{\alpha (I_{1}-3)}}$$

(9) $$P_{VW}=\lambda_{2}\sqrt{\frac{e^{C_{2}\left(I_{1}-3\right)}-\frac{\lambda_{2}^{-2}\lambda_{1}^{-2}}{2}}{1-\frac{\lambda_{2}^{-2}\lambda_{1}^{-2}}{2}}}$$

**Figure 2:**
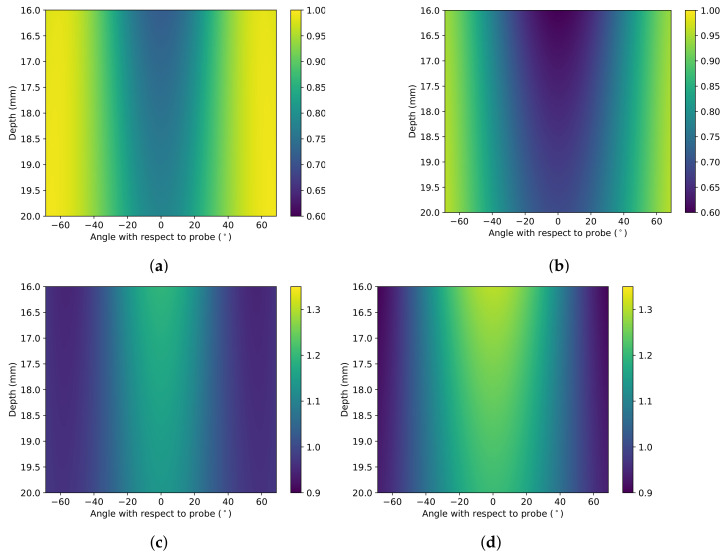

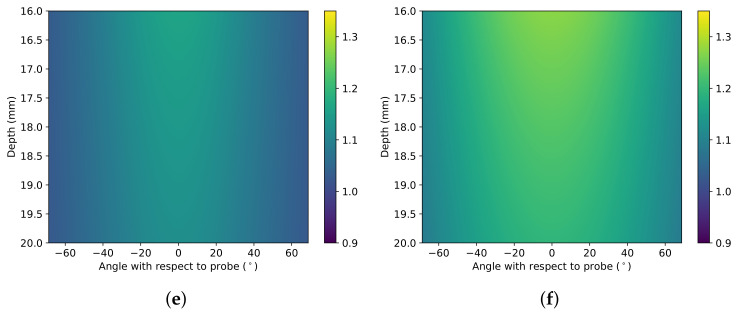
FEBio simulation of $\lambda_{1,2,3}$ values at 8 and 14 mm indentations. (**a**) $\lambda_{1}$ at 8 mm. (**b**) $\lambda_{1}$ at 14 mm. (**c**) $\lambda_{2}$ at 8 mm. (**d**) $\lambda_{2}$ at 14 mm. (**e**) $\lambda_{3}$ at 8 mm. (**f**) $\lambda_{3}$ at 14 mm.

In the simulation, $\lambda_{1}$ is defined as the principal stretch in the direction of the probe indentation. The simulation showed a decrease in the $\lambda_{1}$ and an increase in the $\lambda_{2,3}$ values with increasing indentation. Figure 2 shows the pattern of deformation caused by the probe in polar coordinates.

The maximum change in λ is at the point of contact between the probe and the tissue. The effect spreads out from the point of contact, decreasing in magnitude with distance from the probe. This study defines the area of effect at the different threshold intensities ($AOE_{thres}$) as the number of pixels where the proportional increase from the base speed is above the threshold.

**Figure 3:**
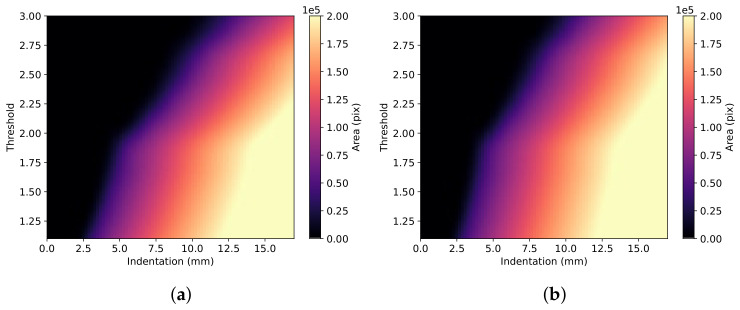
Changes in $AOE_{thres}$ with the threshold value and indentation value for (**a**) DF (α = 5) and (**b**) VW ($C_{2}$ = 5) material models.

The magnitude of change increases with increasing indentation. Figure 3 shows the difference in the areas of varying thresholds with rising levels of indentation. The thresholds used range from 1.10 to 3.00 proportional-increase from the base speed.

**Figure 4:**
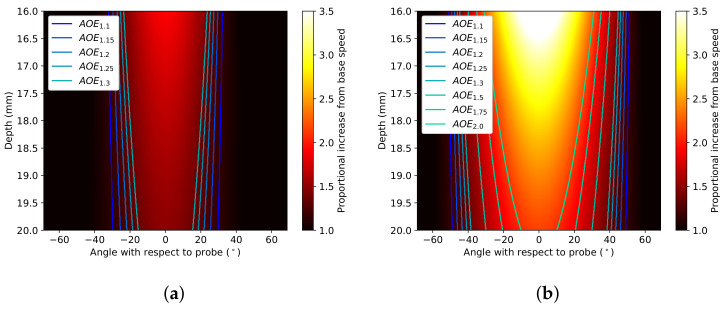
FEBio simulations of the proportional increase in tissue (DF, α=3.2) when the probe is at (**a**) 8 and (**b**) 14 mm indentations. Borders of proportional increase thresholds are marked by contours on the image. Areas within the borders have a proportional increase from base speed higher than stated thresholds.

The simulated tissue was converted from Cartesian to polar coordinates for efficient translation between clinical elastograms and simulated elastograms. The dimensions of each pixel are 0.023${}^{{}^{\circ}}$ and 0.008 mm. Figure 4 shows samples of the simulated proportional-increase field with traced outlines of $AOE_{thres}$.

**Figure 5:**
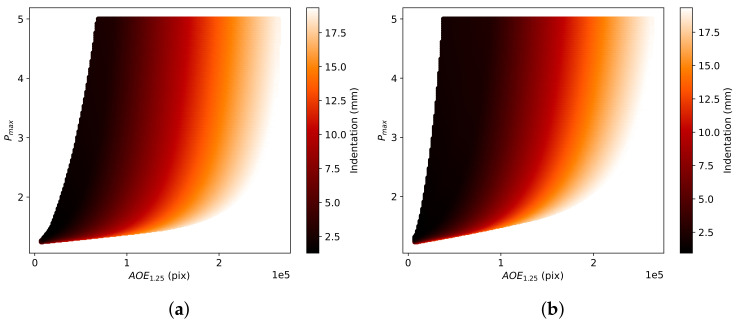
Change in estimated indentation with $AOE_{1.25}$ and $P_{max}$ for (**a**) DF and (**b**) VW material models.

**Figure 6:**
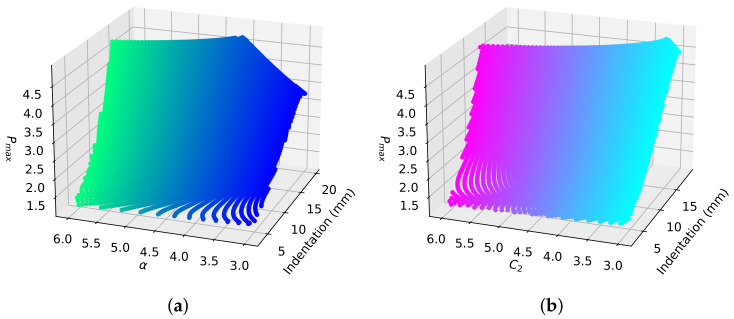
Change in $P_{max}$ with indentation and stiffening parameters α and $C_{2}$ from 3 to 6 for (**a**) DF and (**b**) VW material models, respectively.

Each proportional-increase field is unique to the material model, indentation and stiffening parameter. The area of effect ($AOE_{thres}$) and the peak proportional-increase ($P_{max}$) can be used to define each unique proportional-increase field. This study defines $P_{max}$ as the average proportional increase from the base speed along the central axis from the probe where there is the maximum change in the magnitude of principal stretch. Simulation-based fitting is used to determine the parameters of each elastogram. The values of the measured $AOE_{thres}$ and $P_{max}$ are used to calculate the indentations and stiffening parameters for each material model, as shown in Figure 5 and Figure 6.

The assumptions applied for this simulation are as follows: Firstly, cervical tissue is nearly incompressible. Secondly, the parameters of the tissue are constant within the measured area. Section 6 discusses the problems and limitations of these assumptions.

## 4. Method and Materials

### 4.1. Collection of Data

The data were collected from the Royal Prince Alfred Hospital (RPAH) from November 2020 to August 2021. This study used data from a single cohort of women who came in for standard morphology (18–22 weeks) scans. The study excluded patients with high-risk obstetric history (previous preterm birth, previous cervical surgery) or identification of a short cervix at the morphology scan. Eligible women were informed about the study and asked to consent to a cervical elastography scan. The sonographer used the Aplio 500 machine (Canon Medical Systems, Japan) equipped with a 3–11 MHz transvaginal transducer to make all measurements. The local hospital ethics committee approved the study (Protocol No. X15-0274 and HREC/15/RPAH/375)

**Figure 7:**
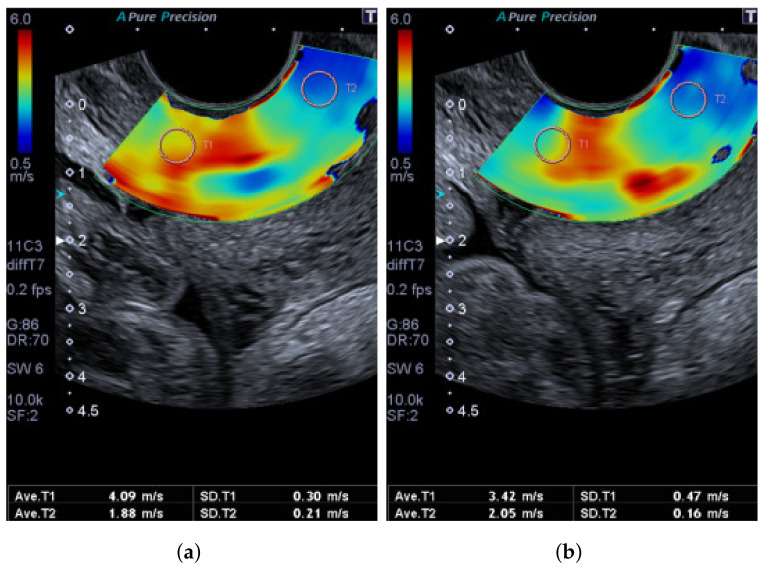
(**a**,**b**) Sample patient elastograms taken with the normal measurement method with minimal pressure applied.

For the standard measures of the shear-wave speed, the sonographer took four elastograms with two measurements in each, an internal os and external os measurement. Two elastograms had minimal applied pressure, and two elastograms had some amount of pressure. The amount of pressure was described as “maximum pressure without causing discomfort to the patient”. A Region of interest (ROI) bubble of diameter 5 mm was placed in an area representing the interior of the cervix and the exterior of the cervix along the anterior lip. This is shown in Figure 7.

### 4.2. Hyperelastic Material Measurement

**Figure 8:**
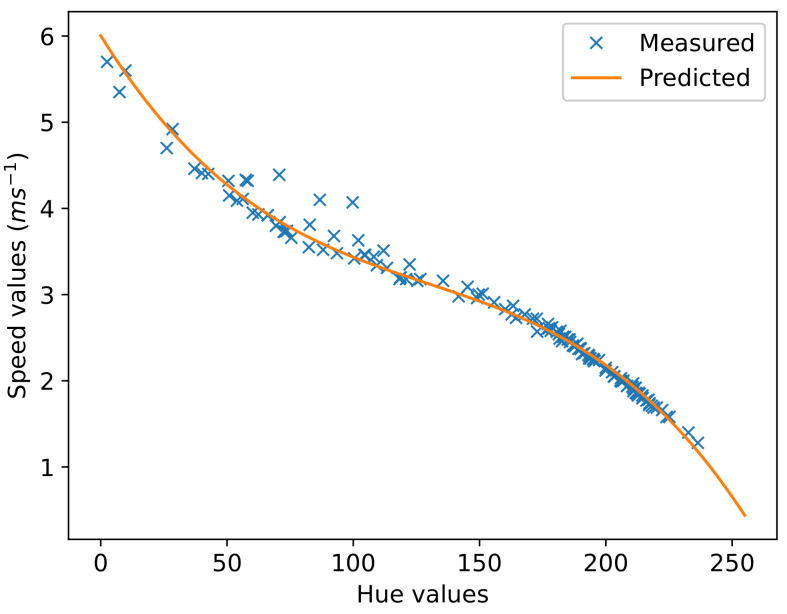
Conversion function of measured hue in image to raw speed value in the elastogram.

For the measurements of hyperelastic material, the sonographer used the same four elastograms without the placement of ROI bubbles. Due to the limitations of the software, the authors could not obtain a raw measurement of the continuous shear-wave speed field. The ultrasound machine does not provide a continuous map of the shear-wave speed values shown. Instead, the measurements were limited to the mean and standard deviation values within the ROI bubbles and not of the entire area chosen. The continuous shear-wave speed field was calculated from the hue of the elastogram. The conversion from hue to shear-wave speed, as shown in Figure 8, is obtained by taking the hue value within an ROI and the corresponding shear-wave speed and fitting it to a material model. The effects of this limitation are further discussed in Section 6.

For consistency, this paper used shear-wave speeds measured between 6 and 10 mm depth from the surface of the probe (16 to 20 mm from the centre of the probe). The area measured needed to be constrained to the anterior lip of the cervix for all elastograms as the presence of the cervical canal disrupts the propagation of shear waves. Furthermore, it is reported that areas too close and too far away from the probe suffer from a lack of resolution [3,5].

**Figure 9:**
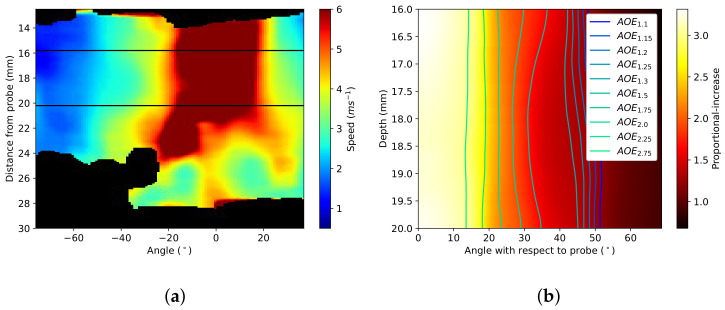
(**a**) Sample elastogram of a patient in polar coordinates with the marked area of interest. (**b**) Sample elastogram with angle measured from the central axis of applied force. Contours on the image mark borders of proportional increase for thresholds from 1.10 to 3.00. Areas within the borders have proportional increase from base speed higher than stated thresholds.

**Figure 10:**
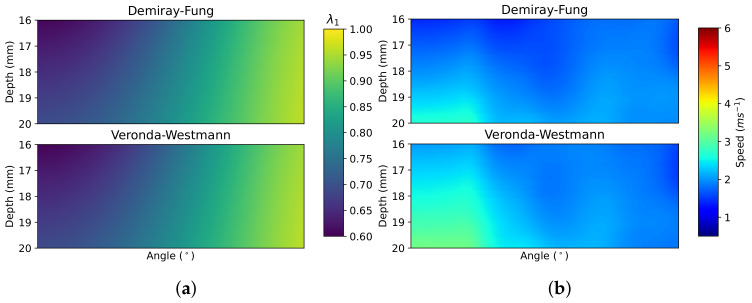
(**a**) Predicted $\lambda_{1}$ values and (**b**) simulated base speed values using predicted material properties for sample elastogram in DF and VW models.

**Figure 11:**
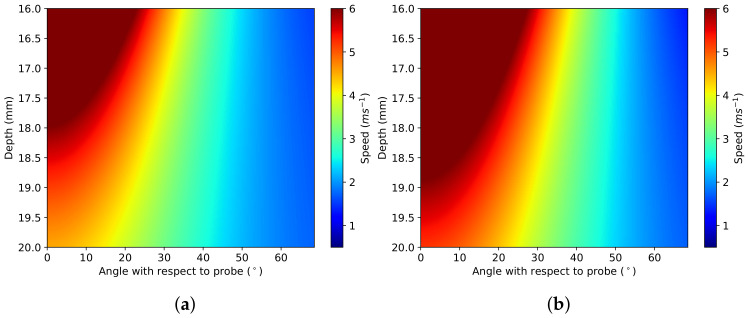
Simulated elastograms for the sample case based on the measured indentation and parameters for the (**a**) DF and (**b**) VW models.

**Figure 12:**
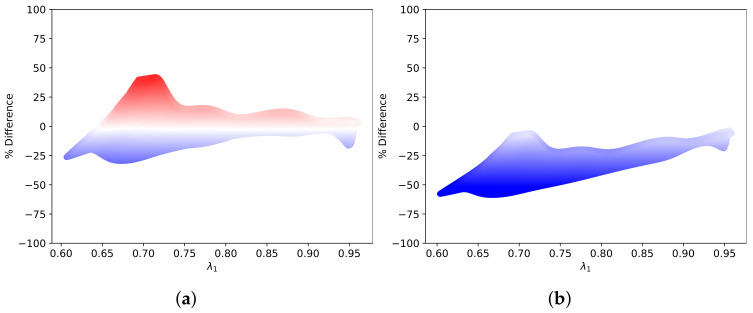
Percentage difference of predicted proportional increase in speed to measured proportional increase in speed with $\lambda_{1}$ for (**a**) DF and (**b**) VW in the sample case.

Figure 9, Figure 10, Figure 11 and Figure 12 demonstrate the process of collecting and estimating the hyperelastic material properties of the cervix for a single case. The shear-wave speed colour map was isolated and converted from Cartesian to polar coordinates, as shown in Figure 9a. The initial base speed estimate was taken as the mean speed between the two elastograms (elastogram with minimum and maximum pressure applied, respectively) in areas with less than 10% difference in the measured shear-wave speed values. The $P_{max}$ of the elastogram was measured, and the effect of the probe was assumed to be symmetrical. From the base speed estimate and the shear-wave speed map, the proportional-increase field is calculated. Figure 9b shows the proportional-increase field and the contours of the thresholds for $AOE_{thres}$ for the sample case.

The method discussed in Section 3 is used to estimate the indentation and stiffening parameters from the measured $AOE_{thres}$ and $P_{max}$ of the proportional-increase field. The predicted indentation and stiffening parameters are used to calculate the theoretical proportional-increase from base speed. The theoretical proportional-increase field is compared to the measured proportional-increase, as shown in Figure 12. The theoretical base speed is calculated by removing the theoretical proportional-increase field from the original elastogram, as shown in Figure 10b. The theoretical base speed is compared to the initial estimate of the base speed. The simulated elastogram was calculated from the theoretical base speed, indentation and stiffening parameter, as shown in Figure 11. The simulated elastogram is compared to the original elastogram using the root mean square deviation (RMSD) measurement between the two images. The similarity between the raw elastogram and the simulated elastogram reflects the simulation’s accuracy.

## 5. Results

### 5.1. Normal Measurement

The demographic information for the cohort is found in Table 3.

Table 4 reports the mean measurements of the internal and external shear-wave speed with and without pressure. The internal measurements were higher than the external measures by 24% without pressure and 38% with pressure. The addition of pressure increased the shear-wave speed measurements of the internal ROI by an average of 27% and the external ROI by 13%. The relative change in speed with pressure is consistent with prior studies comparing internal and external areas of the cervix and its increase with pressure [9].

**Figure 13:**
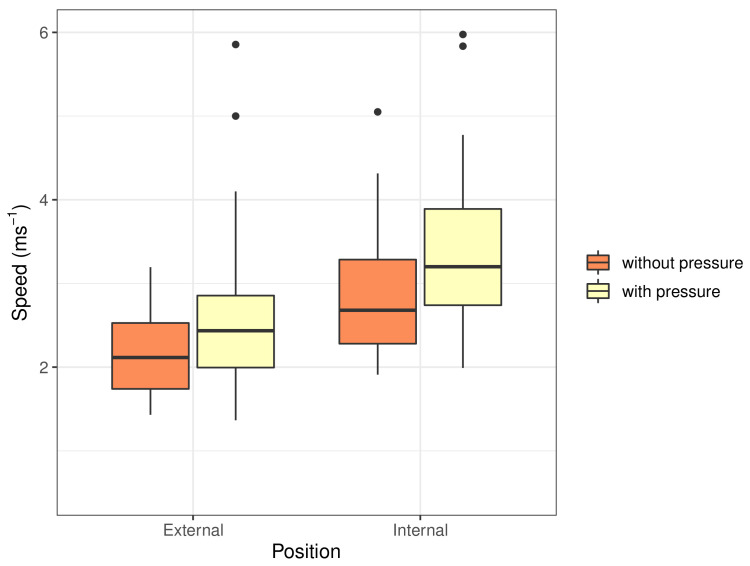
Distribution of measured shear-wave speed values at the internal and external positions with and without pressure.

Figure 13 shows the distribution of the shear-wave speed measurements. The internal measures have a slightly wider distribution than the external measures of speed. Similar measurements are expected as they come from the same low-risk cohort.

**Figure 14:**
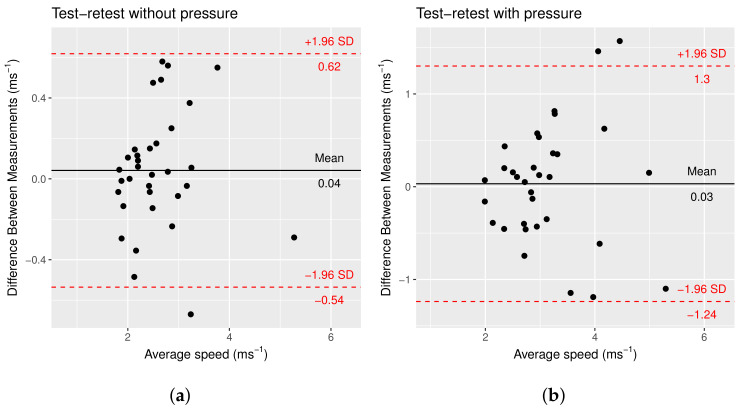
Bland–Altman plot of mean speed in the image for test–retest mean measured speed (**a**) without pressure and (**b**) with pressure. The 95% confidence intervals are marked in red.

The mean shear-wave speed measured for each elastogram is compared for each patient. The mean difference between the two measurements taken at each pressure level was 0.041 ms${}^{-1}$ (95% CI: −0.53, 0.62) without pressure and 0.032 ms${}^{-1}$ (95% CI: −1.23, 1.31) with pressure. The standard deviation of the differences was much higher in the values measured with pressure (0.65 ms${}^{-1}$) than the values measured without pressure (0.29 ms${}^{-1}$). Figure 14 shows the Bland–Altman plot for the test-retest with and without pressure. Based on the Shapiro–Wilk test, the differences are normally distributed for measurements taken without pressure (W = 0.96392, *p*-value = 0.3324) and with pressure (W = 0.97066, *p*-value = 0.4987).

The values measured with pressure are less precise than those measured without pressure. The magnitude of applied pressure is random and thus causes a randomly varying increase in the measured shear-wave speed.

The increase in shear-wave speed is not uniform with the length cervix; it is concentrated at the point of contact. The positioning of the discrete ROI bubbles is another factor that causes measurement errors. If pressure is applied at the internal os, the measured value at the internal os will increase while the value at the external os will remain the same. The change is concentrated such that small shifts in the ROI placement can lead to significant inconsistencies. In addition to problems with changes in shear-wave speed measures, the application of pressure also deforms the cervix. As a result, the ROI needs to be replaced for each elastogram. The difference in positions is an average of 1.47 mm horizontal 1.44 mm vertical for the internal ROI and an average of 1.23 mm horizontal 1.38 mm vertical for the external ROI. The shift in the ROI bubbles may be another source of error in the measurement values.

### 5.2. Hyperelastic Material Measurement

Table 5 summarises the mean predicted indentation and stiffening parameters of the anterior lip of the cervix in a low-risk population. The mean base speeds for the material models were 2.06 and 2.04 ms${}^{-1}$ DF and VW, respectively. This speed is lower than the mean speed without pressure measured in Section 5.1. The difference in speed is likely due to some excess pressure from the probe. The pressure is present even without explicitly applying pressure due to the contact needed for ultrasound imaging. An example of this scenario is shown in the sample case in Figure 7.

**Figure 15:**
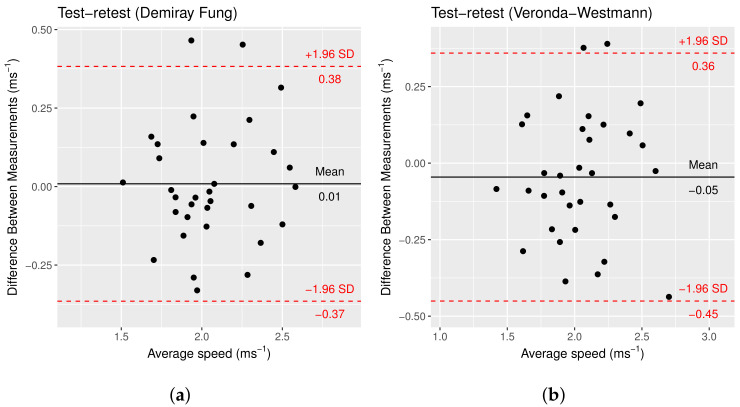
Bland–Altman plot of mean speed in the image for test–retest mean measured speed (**a**) DF and (**b**) VW material models. The 95% confidence intervals are marked in red.

The mean shear-wave speed measured for each predicted elastogram is compared for each material. The mean difference between the two measurements with pressure using the simulation-based method was 0.001 ms${}^{-1}$ (95% CI: −0.37, 0.38) and −0.04 ms${}^{-1}$ (95% CI: −0.45, 0.36) for DF and VW, respectively. The standard deviation of the differences was 0.19 and 0.21 ms${}^{-1}$ for DF and VW, respectively. Figure 15 shows the Bland–Altman plot for the test–retest with pressure with the simulation-based method. The Shapiro–Wilk test shows that the differences are normally distributed for the DF (W = 0.96414, *p*-value = 0.337) and VW (W = 0.98249, *p*-value = 0.8577) material models. The method proposed improves the precision of the measurements made when additional pressure is applied.

**Figure 16:**
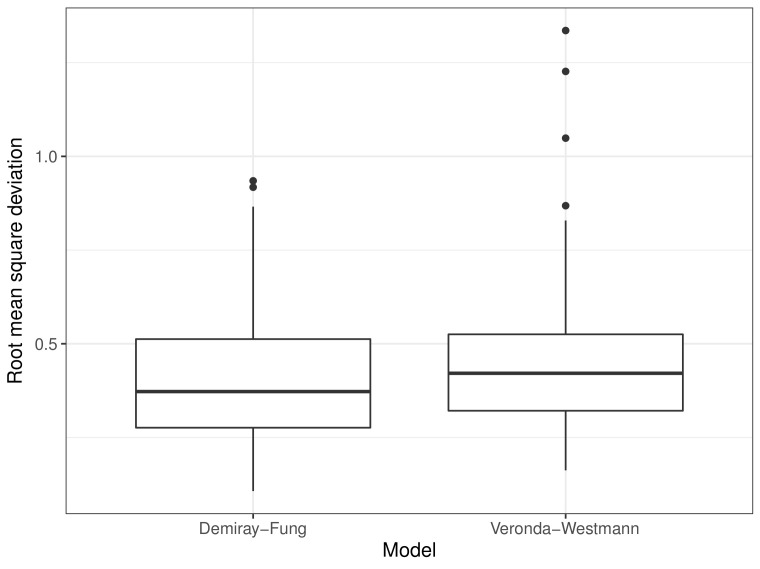
Distribution of root mean square deviation of predicted elastogram with measured elastogram for DF and VW.

The simulated and measured elastograms were compared by measuring the RMSD difference between the images. The average RMSD of the images DF and VW was 0.41 ms${}^{-1}$ (95% CI: 0.38, 0.45) and 0.47 ms${}^{-1}$ (95% CI: 0.43, 0.52), respectively. Figure 16 shows the distribution of the RMSD of the images, which shows DF performed slightly better than VW. For the majority of the cases, the differences were highest in the areas further from the probe at the cervical canal area.

**Figure 17:**
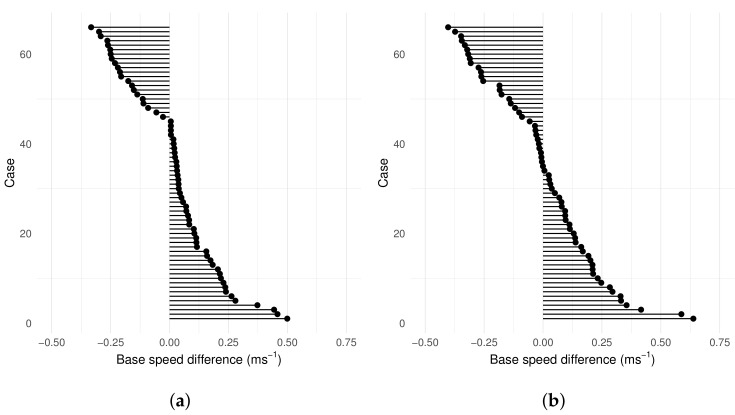
Difference in base speed values estimated and measured for (**a**) DF and (**b**) VW.

Figure 17 compares the theoretical base speed value calculated from the indentation and parameters to the initial base speed estimated from the elastograms for each case. For DF material model estimates, 68% of the cases had a theoretical base speed value higher than the initial base speed estimate. For VW this was 52%. The differences in the base speeds are likely due to the elasticity gradient across the cervix and pre-stress from the surrounding environment. The effects of these factors are discussed in Section 6.

**Figure 18:**
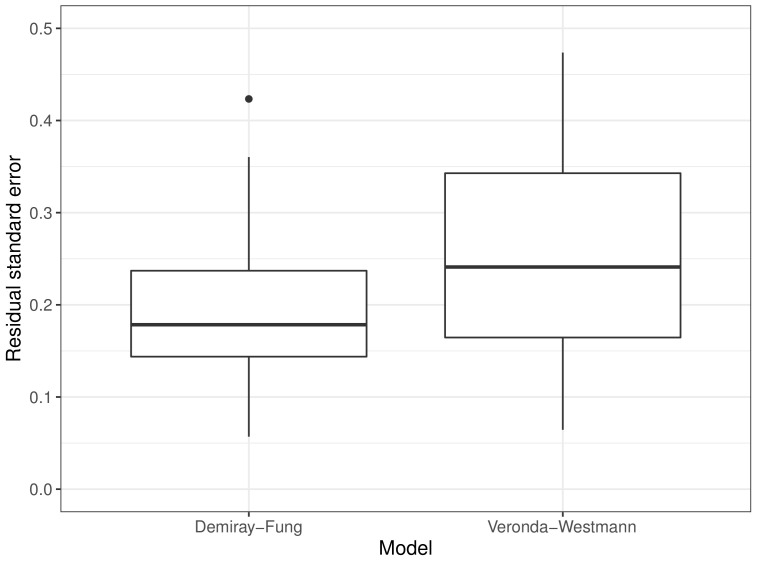
Residual standard error of the predicted proportional increase and the measured proportional increase for DF and VW material models.

The measured proportional increase is compared to the theoretical proportional increase. The residual standard error (RSE) of the proportional increase is a measure of how well the measured value fit the proposed function of the material model. The comparison in percentage difference is demonstrated for the sample case in Figure 12. Figure 18 shows the distribution of the RSE for DF and VW. DF performed marginally better than VW in describing the behaviour of cervical tissue.

## 6. Discussion

This study and many other studies support the theory that pressure on soft tissue changes its shear-wave speed measurement [11,12,13,14,15]. The raw results showed that even when minimal pressure is applied, there are still changes in the measured speed due to the placement of the probe. Figure 7 shows an example of this behaviour. The images are taken with minimal applied pressure, but there is a 0.67 ms${}^{-1}$ change in the measurement taken at the internal os while that of the external os is 0.17 ms${}^{-1}$. The increase in shear-wave speed along the cervix is uneven due to the shape of the probe. The increase in shear-wave speed from the base speed depends entirely on the placement of the probe and the amount of pressure applied. Additionally, there is a shift in the ROI bubble placement when compression is used due to the deformation.

Other studies investigating the nonlinear characteristics of cervical tissue have similarly used hyperelastic materials to describe it. Badir et al. used the aspiration method to characterise cervical tissue throughout the pregnancy [25]. The aspiration method uses suction on a small area of the tissue at the external os. The pressure required to deform the tissue up to 4 mm is measured. Inverse finite element analysis is then used to calculate the neo-Hookean model of the tissue. The study shows a decrease in the neo-Hookean parameter $c_{1}$ from 1.895 to 0.650 kPa from the first to the third trimester. Callejas et al. used uniaxial tension experiments to characterise the properties of ex vivo cervical tissue from hysterectomy patients. The Mooney–Rivlin and Ogden models were used in the study. The results showed that there are distinctly different properties for the epithelium layer and the connective layers of the tissue. The mean parameters of the Ogden model were $\mu_{r}$ = 0.41 MPa, $\alpha_{r}$ = 5.27 and $\mu_{r}$ = 0.94 MPa, $\alpha_{r}$ = 6.40 for the epithelial and connective layer of the cervical tissue, respectively [26].

The estimates made in this study are similar to the measurements made by Badir et al. on a similar population. The results in this experiment measured slightly higher shear modulus measurements, which are expected as the measurements are of the entire cervix, while Badir et al. only made measurements in the external os, which has lower elasticity than the internal os [25]. This study initially considered the neo-Hookean material model. However, it could not fit the high values of the proportional increase caused by the pressure. Measurements from 12/33 patients failed to fit a set of parameters in this case. The neo-Hookean material model is a poor fit because the proportional increase in speed is dependent solely on the principal stretch, as shown in Equations (10)–(12).

(10) $$\Psi_{NH}=C_{1}\left(I_{1}-3\right)$$

(11) $$\frac{\partial \Psi_{NH}}{\partial I_{1}}=C_{1}$$

(12) $$P_{NH}=\lambda_{2}$$

In many of the cases, the rate of change is higher than the principal stretch and is slightly different from patient to patient. Similarly, another study that analysed the nonlinear hyperelastic model fits to brain tissue shear-wave speed data found that neo-Hookean models had the worst fits due to the lack of a stiffening parameter [27]. Therefore, this study excluded the neo-Hookean model. The estimates made in the study by Callejas et al. gave values much higher than the values measured in this study. The differences measured are due to the difference in the loading mechanisms used as soft tissues such as cervical tissue exhibit tension-compression asymmetry.

The method suggested in this study improves the measurement of cervical elasticity. Still, it is not appropriate for use in clinical diagnosis settings as the margin of error is too broad. The measurement errors could be due to several factors. Firstly, the modelled cervical tissue is a simplified model, which ignores some characteristics that it is known to possess. The features include an elasticity gradient from the internal os to the external os and anisotropy in the cervical tissue. The simplified model also ignores pressure effects from the surrounding environment. External sources of pressure cause incorrect estimates of indentation and material properties. Secondly, the method of acquiring raw shear-wave speed data has inaccuracies as it is also an estimate calculated from the colour map. The use of this estimation method is forced by the lack of raw shear-wave speed data from the proprietary software on the SWE equipment. Finally, a limited number of images were collected as the collection process is part of a standard ultrasound imaging procedure at the hospital.

The initial results suggest that to measure the nonlinear properties of cervical tissue, a hyperelastic material model with a stiffening parameter is required. The stiffening parameters of the DF and VW models describe the rate at which the stretch affects the measured shear-wave speed. They are relatively consistent in the data and can be used as additional parameters for diagnosis in future studies. Measurement of the elasticity of the cervix as a continuous structure is more valuable than measuring it with discrete ROI bubbles as it will not be affected by the elasticity gradient across the cervix as well as areas where pressure is concentrated. The shortcomings in this study result from the limited resources and lack of interaction between clinical research and equipment design. The ideal method of quantifying the nonlinear properties of tissue is with measured pressure or strain. For future studies, measured strain using speckle tracking in raw B-mode data is recommended.

The results show that for the nonlinear characteristics of cervical tissue to be effectively measured in a clinical setting, the raw output from the ultrasound machines is required. The proposed method of measuring the strain data would be using the raw B-mode output from the ultrasound machine or tracking the movement of the cervical canal at different pressure levels.

## 7. Conclusions

This study is an initial exploration of hyperelastic material models to replace the existing linear models of tissue representation in elastography. The simulation-based method of measuring the nonlinear characteristics of tissue acquired an approximate baseline of material properties. Applying a general strain map based on the probe shape improved the precision of the measurements. However, a general strain map is not appropriate for adoption as a clinical diagnosis method; a customised strain map is required for each individual case. The approximate nonlinear characteristics of cervical tissue show that models with an exponential increase in shear-wave speed as a function of indentation are necessary to represent cervical tissue with the degree of accuracy required. For future studies, raw data from SWE equipment are needed.

## Figures and Tables

**Table 1:** A summary of strain-energy density functions, and the parameters to be calculated for material models of DF and VW [21,23].

Model	Ψ	$\frac{\partial \Psi }{\partial I_{1}}$	$\frac{\partial \Psi }{\partial I_{2}}$	Parameters
DF	$A_{1}\left[e^{\alpha \left(I_{1}-3\right)}-1\right]$	$A_{1}\alpha e^{\alpha \left(I_{1}-3\right)}$	0	$A_{1},\alpha$
VW	$C_{1}\left[e^{C_{2}\left(I_{1}-3\right)}-1\right]-\frac{C_{1}C_{2}}{2}\left(I_{2}-3\right)$	$C_{1}C_{2}e^{C_{2}\left(I_{1}-3\right)}$	$-\frac{C_{1}C_{2}}{2}$	$C_{1},C_{2}$

**Table 2:** Elements in FEBio simulation.

Part	Element Type	Number of Elements
Probe (rigid)	4-node linear tetrahedral element	3998
Tissue	8-node trilinear hexahedral element	25,600

**Table 3:** Demographics of the study population.

		N = 33 Value [Mean (SD)]
Maternal age (years)		32.76±4.10
Gestational age at test (weeks)		21.32±0.75
Gravidity ( N )	1	26
	2	4
	≥ 3	3

**Table 4:** Median (range) measurements of the shear-wave speed measured using the normal method for the internal and external regions of the cervix.

Measurement	Internal (ms${}^{-1}$)	External (ms${}^{-1}$)	Mean (ms${}^{-1}$)
Speed (No pressure)	2.86 ± 0.75	2.30 ± 0.94	2.58 ± 0.69
Speed (Pressure)	3.63 ± 1.26	2.64 ± 0.94	3.14 ± 0.81

**Table 5:** Median (range) indentation and parameter estimates for DF and VW material models.

Material	Indentation (mm)	Parameter
Demiray–Fung	7.10 (3.81–9.69)	Aα = 2.07 (1.65–2.58) kPa, α = 6.74 (4.07–19.55)
Veronda–Westmann	7.19 (3.90–10.56)	$C_{1}C_{2}$ = 4.12 (3.24–5.04) kPa, $C_{2}$ = 4.86 (2.86–14.28)

## Data Availability

The data is available on request.

## Abbreviations

The following abbreviations are used in this manuscript:

DF	Demiray–Fung
VW	Veronda–Westmann
SWE	Shear-wave Elastography
os	orifice
CI	Confidence Interval
RMSD	root mean square deviation
RSE	residual standard error
ROI	Region of interest
RPAH	Royal Prince Alfred Hospital
